# Gene–Diet Interactions in Colorectal Cancer: Survey Design, Instruments, Participants and Descriptive Data of a Case–Control Study in the Basque Country

**DOI:** 10.3390/nu12082362

**Published:** 2020-08-07

**Authors:** Iker Alegria-Lertxundi, Carmelo Aguirre, Luis Bujanda, Francisco J. Fernández, Francisco Polo, José M. Ordovás, M. Carmen Etxezarraga, Iñaki Zabalza, Mikel Larzabal, Isabel Portillo, Marian M. de Pancorbo, Leire Palencia-Madrid, Koldo Garcia-Etxebarria, Ana M. Rocandio, Marta Arroyo-Izaga

**Affiliations:** 1 Department of Pharmacy and Food Sciences. Faculty of Pharmacy, University of the Basque Country UPV/EHU, 01006 Vitoria-Gasteiz, Spain; lertxundi83@gmail.com (I.A.-L.); anamaria.rocandio@ehu.eus (A.M.R.); 2 Pharmacovigilance Unit, Galdakao-Usansolo University Hospital, Osakidetza, 48960 Galdakao, Spain; carmelo.aguirregomez@osakidetza.eus; 3 Department of Gastroenterology, Donostia University Hospital/Biodonostia Health Research Institute, University of the Basque Country UPV/EHU, Centro de Investigación Biomédica en Red de Enfermedades Hepáticas y Digestivas (CIBERehd), 20014 San Sebastian, Spain; luis.bujandafernandezdepierola@osakidetza.eus; 4 Department of Gastroenterology, Galdakao-Usansolo University Hospital, Osakidetza, 48960 Galdakao, Spain; franciscojavier.fernandezfernandez@osakidetza.eus; 5 Department of Gastroenterology, Basurto University Hospital, Osakidetza, 48013 Bilbao, Spain; franciscopolo2001@gmail.com; 6 Nutrition and Genomics Laboratory, Jean Mayer Human Nutrition Research Center on Aging, Tufts University, Boston, MA 02111, USA; Jose.Ordovas@tufts.edu; 7 Institute IMDEA Food, Institutos Madrileños de Estudios Avanzados (IMDEA), 28049 Madrid, Spain; 8 Department of Pathology, Basurto University Hospital, Osakidetza, 48013 Bilbao, Spain; mariacarmen.etxezarragazuluaga@osakidetza.eus; 9 Department of Physician and Surgeon Specialities, University of the Basque Country UPV/EHU, 48940 Leioa, Spain; 10 Department of Pathology, Galdakao-Usansolo University Hospital, Osakidetza, 48960 Galdakao, Spain; ignacio.zabalzaestevez@osakidetza.eus; 11 Department of Pathology, Donostia Hospital/Biodonostia Health Research Institute, Centro de Investigación Biomédica en Red de Enfermedades Hepáticas y Digestivas (CIBERehd), 20014 San Sebastian, Spain; josemiguel.larzabalaramberri@osakidetza.net; 12 Central Coordinating Center of the Bowel Cancer Screening Program, Healthcare subdirectorate, Osakidetza, 48011 Bilbao, Spain; mariaisabel.portillovillares@osakidetza.eus; 13 BIOMICs Research Group, University of the Basque Country UPV/EHU, 01006 Vitoria-Gasteiz, Spain; marian.mdepancorbo@ehu.eus (M.M.d.P.); leire.palencia@ehu.eus (L.P.-M.); 14 Biodonostia Health Research Institute, Gastrointestional genetics group, Centro de Investigación Biomédica en Red de Enfermedades Hepáticas y Digestivas (CIBERehd), 20014 San Sebastian, Spain; koldo.garcia@biodonostia.org

**Keywords:** colorectal cancer, diet, genetic factors, gene–diet interactions, risk-factors, case–control study

## Abstract

Epidemiologic studies have revealed inconsistent evidence of gene-diet interaction in relation to colorectal cancer (CRC). The aim of this study was to analyze them in a sample of cases and controls from the population-based bowel cancer screening program of the Osakidetza/Basque Health Service. This study analyzed dietetic, genetic, demographic, socioeconomic factors and lifestyles. In the present manuscript, the survey design, sampling, instruments, measurements and related quality management were presented. Moreover, we analyze differences between cases and controls in some data, especially those related to diet. The participants were 308 cases and 308 age- and sex-matched subjects as controls. Cases were more likely than controls to have overweight/obesity (67.5% vs. 58.1%, *p* < 0.05), a lower intake of vitamin B_2_ (0.86 ± 0.23 vs. 0.92 ± 0.23 mg/1000 kcal, *p* < 0.01) and calcium:phosphorus ratio (0.62 ± 0.12 vs. 0.65 ± 0.13, *p* < 0.01). A higher proportion of cases than controls did not meet the Nutritional Objectives for saturated fatty acids (85.7% vs. 67.5%, *p* < 0.001) or cholesterol (35.4% vs. 25.0%, *p* < 0.01). In conclusion, the present study provides valuable data for analyzing the complexity of gene-diet interaction in relation to CRC. The results presented here suggest that overweight/obesity and a high intake of certain dietary components, especially saturated fatty acids and cholesterol, are more frequent in cases than in controls.

## 1. Introduction

Colorectal cancer (CRC) is already the third leading cause of cancer death in the world, and its incidence is steadily rising in western countries [1]. According to GLOBOCAN 2018 data, CRCs are the third most commonly diagnosed form of cancer globally, comprising 11% of all cancer diagnoses [2]. Incidence varies geographically; more-developed regions have a higher incidence than less-developed ones [3]. Europe is among the seven world regions ranked according to increasing age-standardized incidence rate (ASRi) with an ASRi of 30.0 per 100,000 [4]. In particular, in Spain, the ASRi for CRC is 33.4 per 100,000 (21.1 per 100,000 in males and 12.4 per 100,000 in females) [5]. In the Basque Country, one of the autonomous regions of the North of Spain, this pathology is the most frequent type of cancer (taking into account the combined incidence of both sexes) [6].

During recent years, mortality rates for CRC have been decreasing due to early screening programs [7,8] and better treatment options [9]. However, the etiology of CRC is complex and still not fully understood. Both genetic and environmental factors play an important role in the etiology of this disease [10]. Large population studies with varying strength of evidence have found CRC-protective factors such as certain food groups (fruits and vegetables [11], fish [12]), specific foods (garlic [13] and coffee [14]) and dietary components (fiber, resistant starch, folate, vitamin B6, vitamin D, calcium and magnesium) [9,15]. In addition, physical activity (PA) [16] and certain drugs (aspirin, non-steroidal anti-inflammatory drugs [17] and hormonal replacement therapy (HRT) in postmenopause [18]) could decrease the CRC risk. However, red/processed meat [19], tobacco and alcohol consumption [20] and obesity [21] are considered risk factors for CRC.

In any case, protective and/or risk factors are not present in isolation, but coexist and interact with each other and with other factors; for example, both dietary and genetic factors affect CRC risk in an interactive manner [22]. The recognition of these gene–diet interactions as a driver in CRC may open up new areas of research in disease epidemiology, risk assessment and treatments. To date, epidemiologic studies have revealed inconsistent evidence of gene–diet interaction in relation to CRC. In order to better elucidate the role of these types of interactions in the etiology of CRC, the aim of this study was to analyze them in a sample of cases and controls from the population-based bowel cancer screening program (BCSP) of the Osakidetza/Basque Health Service. The main advantage of the present study compared to other similar research [23,24,25] is that we confirmed that controls were free of the disease through colonoscopy. Colonoscopy was used as the diagnosis criterion to identify the cases in order to avoid false positives and negatives.

In the present manuscript, the survey design, sampling, instruments, measurements and related quality management were presented. This detailed information will allow its replication in other populations for a comparison of the results. In addition, we analyze differences between cases and controls in some data, especially those related to diet, but also in demographic data, weight status, lifestyle (different from diet), quality of life and stress level and use of drugs related to decreasing CRC risk.

## 2. Materials and Methods

Overall, this epidemiologic study is an observational analytic case-control study designed to address possible gene–diet interaction in relation to CRC.

### 2.1. Sampling and Study Subjects

Participants in this study were recruited from among patients attending any of the three hospitals of the Osakidetza/Basque Health Service (Basurto, Galdakao and Donostia) members of the Basque Country’s BCSP. To be eligible for this BCSP, the patients had to be aged between 50 and 69, asymptomatic for colorectal symptoms and registered with the Osakidetza/Basque Health Service [26]. These inclusion criteria were applied to both case and control groups; that is, controls fulfilled the same eligibility criteria defined for the cases, with the exception of the disease (outcome). The sample size was estimated to be 286 in each group to detect an odds ratio (OR) of 2.0 with 80% power at a two-sided level of significance of 5%, under an exposure prevalence of 10%, using the Epidat 3.0 program (Dirección Xeral de Saúde Pública, Xunta de Galicia & Organización Panamericana de la Salud. Santiago de Compostela, A Coruña, Spain, 2004). Recruitment and data collection through questionnaires were conducted between 2014 and 2016. The start date of the study was 2014 because the BCSP in the Basque Country reached the whole target population (approximately 586,700 people) at the beginning of this year [26].

All the patients who were newly diagnosed with CRC (*n* = 601) were invited to participate in this study. Of those, 283 refused to participate in the study, and 10 were excluded due to missing information. Ultimately, 308 subjects (66.2% men) consented to participate in the survey and completed all the questionnaires. In addition, for each case, three age- (±9.0 years) and sex-matched control patients were randomly sought from the list of CRC-free subjects (*n* = 1836) who participated in the BCSP during the same period as the cases. The matched controls were patients with positive results (abnormal) for immunochemical fecal occult blood test (iFOBT) and negative colonoscopy results (normal). The participation rate of the controls was 37.6%, and 17 subjects were excluded due to missing information. Finally, the matched case-to-control ratio was 1:1, and the final data set included 308 cases who were diagnosed with CRC and 308 age- and sex-matched controls. In those cases for which there was more than one control per case, one was randomly selected for analysis. Further details on recruitment and data collection have been described elsewhere [27].

The characteristics of the cases (pathological staging, location of the cancer, tumor grade and treatments) have been also described before [28]. Briefly, 72% were diagnosed with early-stage (I/II) CRC, 76% had distal location of the cancer, 80.5% of tumors were well-/moderately differentiated and 73.7% had undergone surgical resection. The cases were invited to take part in this survey at least one month after finishing their last treatment (surgery, chemotherapy or radiotherapy) (median, 1.3 years; range, 0.1 to 4.2 years). No statistically significant differences were found in the time elapsed between participation in the BCSP and collaboration in this survey, between cases and controls (cases, 1.8 ± 1.0 years, controls 1.6 ± 1.5 years; *p* > 0.05). All these clinical data were obtained from the Basque Country’s population-based BCSP database, which links patient medical records and clinical databases and was reviewed by expert staff. This study was conducted according to the guidelines laid down in the Declaration of Helsinki, and all procedures involving patients were approved by the Clinical Research Ethics Committee of the Basque Country (reference numbers PI2011006 (approval date: 03/23/2012) and PI2014042 (approval date: 05/28/2014). Written informed consent was obtained from all the study participants.

### 2.2. Procedures and Survey Modules

The modules in this study were initially selected to cover the assessment of dietary intake, lifestyle, demographic and socioeconomic determinants and genetic factors. Table 1 provides an overview of the major study components. Consenting participants self-completed and returned one general questionnaire (GQ) and a short Food Frequency Questionnaire (SFFQ). The questions referred to behaviors before participating in the BCSP. Assistance from the study staff was available to help the patients to understand the items on the questionnaires.

#### 2.2.1. General Questionnaire

The GQ was used to gather information on weight status (self-reported weight and height), environmental factors (demographic and socio-economic factors: age and sex, marital status and children, birthplace, place of residence, total number of co-residents per household, total number of rooms (excluding the kitchen and bathrooms), educational attainment, economic activity and last work; and lifestyle information: PA, physical exercise (PE) and smoking consumption). These questions were taken from the Spanish Health Questionnaire [25]. Body mass index (BMI), estimated from self-reported height and weight, was classified according to the WHO criteria for those under 65 years of age [29] and according to the criteria proposed by Silva Rodríguez et al. for those 65 and older [30].

The GQ included as well information about perceived quality of life (QoL) and stress, and the use of drugs related with decreasing CRC risk (antiplatelet (including non-steroidal anti-inflammatory drugs), anticoagulants and HRT in the case of women) [31,32,33,34]. To assess the QoL and perceived stress, an analog linear scale with a range from 0 to 100 was used [35].

The differences in general characteristics (age, BMI, educational attainment, economic activity, last employment, PE and smoking habit) between cases and controls were previously described (World Journal of Gastroenterology, in review process). Briefly, significant differences between cases and controls were found for educational level, smoking and weight status, with a higher percentage of cases with low–medium educational level, past or current smoking status and with overweight/obesity compared to controls (*p* < 0.01).

#### 2.2.2. Dietary Habits Questionnaire

Diets were assessed using a self-reported SFFQ that was a modified version of the Rodríguez et al. [36] questionnaire. This adaptation was validated with multiple 24-h recalls in the Basque general population [37] and in CRC-diagnosed patients in a pilot of the present study [38]. It consisted of 67 items and requires the subjects to recall the number of times each food item was consumed either per week or per month. This SFFQ included specific questions about frequency of intake of alcoholic beverages. Moreover, the respondents could also record the consumption of other foods that were not included on the food list, as well as the use of dietetic products and nutritional supplements (generic and brand-name, dose and frequency). The SFFQ included additional items to ask about the consumption of fried foods, grilled or roast meat, added salt (cooking and at the table), as well as the average weekly consumption of some food types (cooked vegetables, salads, fruit/fruit products, fish/fish products and meat/meat products/meat dishes). These last questions were taken from the EPIC-Norfolk Food Frequency Questionnaire [39,40].

Once the completed SFFQ was received, it was reviewed by a dietitian. Consumption frequencies were standardized to “per day” and multiplied by standard serving sizes (grams) [41]. For items that included several foods, each food’s contribution was estimated with weighting coefficients that were obtained from the usual consumption data [42]. Food items were then regrouped according to nutritional characteristics [43] and considering the potential contribution of food to the pathogenesis of CRC [44,45]. All food items that were consumed were entered into DIAL 2.12 (2011 ALCE INGENIERIA) [46] a type of dietary assessment software, to estimate energy intake (kilocalories/day, kcal/d), nutrients and dietary compounds intakes that were expressed in absolute values, as a percentage of the total energy intake (TEI) and as daily consumption per 1000 kcal.

Some nutrients and dietary compounds intakes were estimated by other food composition databases as detailed below. Methyl donor compounds (methionine, choline and betaine), fatty acids (arachidonic, eicosapentaenoic and docosahexaenoic) and dietary antioxidants (pro-vitamin A carotenoids (α-carotene, β-carotene and β-cryptoxanthin) and lutein) were estimated using the US nutrients database [47]. In addition, flavonoids and lignans intakes were calculated using Phenol-Explorer [48].

Mean daily energy intake was compared with energy requirements derived from basal metabolic rate that was estimated using the Harris–Benedict formula [49] multiplied by activity factors [50]. The activity factor was based on self-reported main daily activity. Nutrient intake data (from diet and dietetic products and supplements) were compared with the Nutritional Objectives for the Spanish Population (NOSP) [51], the estimated energy requirements (EER), the estimated average requirements (EAR) or adequate intakes (AI) [52,53]. The EAR is the mean daily intake value, which is estimated to meet the requirement of half of healthy individuals in a life-stage and sex group for that nutrient, and the AI is established when there is insufficient scientific evidence to determine an EAR [52,53]. Results of energy and micronutrients were expressed as a percentage of the EER and EAR or AI, respectively. Micronutrient data were also compared with Tolerable Upper Intake Levels (UL) [54,55,56,57,58]. Moreover, caffeine consumption was compared to Denmark’s and the UK’s recommended limit for caffeine intake [59].

Additionally, in this survey, we calculated a mineral score, which is a modified version of the score proposed by Swaminath et al. [60] who have associated this score with CRC risk. In the present study, this mineral score included six minerals with possible colon anti-carcinogenic effects (calcium, magnesium, zinc, selenium, potassium and iodine) and four with pro-carcinogenic properties (iron, copper, phosphorus and sodium). We did not include intakes of manganese, even though it was part of the original score, due to the lack of data on this mineral in the food composition database used in the present study. Mineral intake was expressed as daily consumption per 1000 kcal, and then the intakes of each mineral were categorized into tertiles based on the distribution within the controls group (taking into account sex differences when they were significantly different).

We applied a similar score methodology to that developed by Swaminath et al. [60]. That is, for each mineral hypothesized to reduce CRC risk, each participant was assigned a value equal to their tertile rank (i.e., a value of 1–3, with lower ranks indicating lower mineral intakes and higher ranks indicating higher mineral intakes). For each mineral hypothesized to have predominantly pro-carcinogenic properties in the colon, the values assigned to the rankings were reversed (i.e., values of 3–1, with lower ranks indicating higher mineral intakes and higher ranks indicating lower mineral intakes). Finally, each participant’s values for each mineral were summed to represent his/her mineral score; thus, the range of possible scores was 10–30.

Regarding alcohol consumption, the SFFQ used in this study included specific questions about the frequency of intake of the following five major types of alcoholic beverages: beer, wine, cider, aperitif with alcohol and liquor. The alcohol consumption data were expressed as grams of alcohol and standard drink units (SDU) per week [61]. We used the SDU defined for Spain (one SDU is the equivalent to 10 g of alcohol). With this information, the participants were categorized into those who did and did not meet the recommendations [62]. Finally, adherence to the dietary recommendations was evaluated utilizing the Healthy Eating Index for Spanish Diet (HEISD) [63] and the MedDietScore (MDS) [64], as previously explained [38]. The theoretical range of the HEISD is 0–100 and of the MDS 0–55; higher values of these scores indicate greater adherence to the dietary recommendations for the Spanish population and the Mediterranean diet pattern, respectively. These results were previously described [28].

#### 2.2.3. Adherence to Guidelines for CRC Prevention

Adherence to guidelines for CRC prevention was assessed using a modified version of the World Cancer Research Fund/American Institute for Cancer Research (WCRF/AICR) score [65]. Of the seven recommendations (components) (six in men) included in the original version of this score, we selected five with convincing evidence of an association with CRC risk [15]: PA/PE, dietary fiber, red meat and processed meat, alcoholic beverages and body fatness (as BMI). The recommendation on abdominal fatness and factors that lead to greater adult attained height were not included in this score because sufficient data were not available.

The score assigned for each component was 1 when the recommendation was met. An intermediate category (0.5 points) was created to appraise a higher proportion of the variability in the population. All other individuals received 0 points. The score was constructed using quantitative criteria laid down in the recommendations as cut-off points WCRF/AICR, and therefore, higher scores indicate a greater concordance with the recommendations of the WCRF/AICR [15]. Moreover, each component of the score was added to calculate a total score for each study participant. The theoretical range was between 0–5 points for both sexes.

#### 2.2.4. Lifestyle Changes after Participating in the BCSP

A questionnaire developed ad hoc that included items related to the recommendations with convincing evidence of an association with CRC risk [15] was used to assess lifestyle changes after participating in the BCSP. This tool was applied to a subsample of 102 matched case-control pairs, randomly selected from the study sample through phone interviews by previously trained health professionals. The questionnaire included the following items: PA/PE, food consumption (vegetables, fruit, whole cereals, red meat, processed meat, alcoholic drinks) and smoking habits.

Possible answers were: “increase”, “decrease”, “the same” or “do not consume/practice”. If the answer was “increase” or “decrease”, additionally, subjects were asked about the reason/s for the change. Answers about the reasons were open and were analyzed manually and categorized as follows: “health promotion”, “food preference”, “changes in work, family or personal life”, “diagnostic and/or side effects of CRC treatment/s”, “other pathologies” and “do not know or missing”.

#### 2.2.5. Data Obtained From Clinical Databases

Data about diagnosis and treatment of cases, as previously mentioned (pathological staging, location of the cancer, tumor grade and treatments), were obtained from clinical databases. Additionally, in both cases and controls, socio-economic level and health status (specifically health resource consumption) data were assessed with two indices that were obtained from the clinical databases developed by the Health Department of the Basque Government, namely the socioeconomic deprivation index (DI) and predictive risk modeling (PRM), respectively. The first one was estimated using the MEDEA project criteria [66], as has been described elsewhere [27], and was divided into quintiles (Q), with the first being the least disadvantaged and the fifth being the most disadvantaged. The DI was successfully assigned to 80.2% of participants, while the quality of the registered information did not permit the linking of the remaining 19.8%. The PRM is an index that is based on Adjusted Clinical Groups (ACG) [67], Diagnostic Cost Groups/Hierarchical Condition Categories (DCG-HCC) [68] and Clinical Risk Groups (CRG) [69]. This index combines information about diagnoses, prescriptions, previous costs and the use of specific procedures. It is capable of predicting the use of health resources [70], and it was stratified into four levels (L): the first included participants with a risk of high health resource consumption, and the fourth included those with low health resource consumption. The PRM was successfully assigned to 95.1% of participants, while the quality of the registered information did not permit the linking of the remaining 4.9%.

The differences in these two indices (DI and PRM) between cases and controls were previously described (World Journal of Gastroenterology, in review process). Briefly, significant differences between the cases and the controls were found for DI and PRM, with a higher percentage of controls than cases in Q1-3 (the least disadvantaged) for DI, and a higher percentage of cases than controls in L1-2 (these levels included those with a risk of high health resource consumption) for PRM (*p* < 0.001).

#### 2.2.6. Biological Samples and Genotyping

In this survey, healthy tissues or saliva samples of 230 CRC patients and 230 controls were collected and genotyped. Samples were provided by the Basque Biobank for Research-OEHUN www.biobancovasco.org and were processed following standard operation procedures with appropriate ethical approval. DNA was extracted using AllPrep DNA/RNA kit (Qiagen, Hilden, Germany) for paraffin-embedded tissue samples and AutoGenFlex Tissue DNA Extraction kit (Autogen, Holliston, MA, USA) for mouthwash saliva samples, and then the extracted DNA was assessed with NanoDrop™ Spectrophotometer (ThermoFisher, Waltham, MA, USA).

Double-stranded DNA was quantified by fluorometry using Quant-iT™ PicoGreen1 dsDNA Assay Kit (Invitrogen, Paisley, UK) on a DTX 880 Multimode Detector (Beckman Coulter, Fullerton, CA, USA) to normalize DNA concentration. SNPs were selected for analysis on the basis of published studies concerning: (1) SNPs associated with susceptibility for development of CRC [71,72] and (2) associations between SNPs and food groups or dietary factors as well as gene–diet interactions in CRC. SNPs were organized in the context of the gene(s) at or near locus and chromosome locus. The allelic discrimination was assessed using the MassARRAY System (Agena Bioscience, San Diego, CA, USA) on CeGen-PRB2-ISCII (Nodo USC–Universidade de Santiago de Compostela, A Coruna, Spain) following the procedure provided by the manufacturer. Quality control samples were included in the genotyping assays.

Regarding the susceptibility SNPs for CRC, after an updated summary of the published SNPs [71,72], 48 previously reported CRC-susceptibility SNPs were selected and analyzed. The results of these SNPs were described in the manuscript of Alegria-Lertxundi et al. [27]. In summary, we have confirmed a CRC susceptibility locus and the existence of associations between modifiable factors and the rs6687758 SNP; moreover, the genetic risk score (GRS) was associated with CRC. In relation to the SNPs associated with food groups or dietary factors as well as gene–diet interactions in CRC, after a bibliographic review on the topic, 82 SNPs were selected and analyzed. A systematic bibliographic review was conducted from January 2000 to May 2015 on the following databases: PubMed/Medline, EMBASE, Scopus, Web of Science, ScienceDirect, Cochrane Library, Google Scholar and Conference Proceedings databases. The search strategy used combinations of the following terms: colorectal cancer, colon cancer, rectal cancer, gene, polymorphism, diet and nutrient. The following inclusion criteria were used for selecting the studies: (1) epidemiological study with a case-control or cohort study design, (2) measurement of CRC incidence, (3) measurement of dietary factors, and (4) assessment of the interaction effect of dietary factors and genetic variants on CRC risk. The identified studies were screened by two researchers independently. The genes included in the study were: *MTRR* (9 SNPs), *MTHFR* (8 SNPs), *MTHFD1* (5 SNPs), *NOS2A* (15 SNPs), *NOS3A* (2 SNP), *SEPP1* (8 SNPs), *EPX* (6 SNPs), *CASR* (10 SNPs), *GATA3* (3 SNPs), *NAT2* (3 SNPs), *IL10* (2 SNPs), *DNMT3B* (10 SNPs) and *MPO* (1 SNP). These results are currently under review.

### 2.3. Quality Management

We applied a similar methodology of those used in the IDEFICS study [73]. A unique subject identification number was attached to each recording sheet, questionnaire and sample, as in other researches. The identification number had to be entered twice before the document could be entered into its respective database. All data were entered twice independently, and deviating entries were corrected. Inconsistencies that were identified by additional plausibility checks were rectified.

### 2.4. Timeline

Consenting participants self-completed and returned questionnaires between 2014 and 2016. Interviews to obtain data about lifestyle changes after participating in the BCSP were carried out between 2015 and 2016 in both cases and controls after self-reported questionnaires were returned. The collection of data from clinical databases, as well as the obtaining of biological samples, was done after receiving and reviewing the questionnaires.

### 2.5. Statistical Analysis

Statistical analyses for the present paper were performed using IBM SPSS Statistics for Windows, version 22.0 (IBM Corp., Armonk, NY, USA). Categorical variables are shown as a percentage, and continuous variables are shown as the means and standard deviation (SD). Normality was checked using the Kolmogorov–Smirnov–Lilliefors test. Paired t-test or the Wilcoxon rank-sum test was used to two related means comparison. The categorical variables were analyzed using the χ^2^ test or the Fisher exact test. The differences between cases and controls in the mineral score were assessed as a continuous variable using Wilcoxon rank-sum and a categorical variable according to tertiles using the χ^2^ test. All tests were two-sided, and *p*-values less than 0.05 were considered statistically significant. However, the threshold for statistical significance was set on the basis of Bonferroni correction for the number of variables related to diet (energy and nutrients intake) at *p* = 0.0016 (0.05 ÷ 31).

## 3. Results

Table 2 provides information regarding the demographic data, weight status, lifestyle (such as main daily activity, alcohol consumption, dietetic products and supplement use), drugs use, and quality of life and stress level. It should be noted that cases had a significantly higher prevalence of overweight/obesity than controls (*p* < 0.05). Although the percentage of controls whose main daily activity was sedentary (sitting or standing) was higher compared to cases, these differences were not statistically significant. In addition, the use of dietary products and dietary or nutritional supplements was similar in cases and controls, with vegetable drinks being the most common dietary product used and mineral supplements the most common nutritional supplement used. On the other hand, the use of antiplatelet agents was more frequent in cases than controls (*p* < 0.05).

With respect to the weekly consumption of some food types, there were no significant differences between cases and controls in intakes of cooked vegetables, salads, fruit/fruit products, fish/fish products and meat/meat products/meat dishes, neither in the consumption of fried foods or grilled or roast meat nor in the frequency of use of salt added at the table. However, a higher proportion of cases “always” or “often” used salt for cooking (76.8%) compared to controls (69.7%) (*p* < 0.05). In any case, the average intake of Na from SFFQ was similar in cases and controls (Table 3).

Table 3 shows the daily energy and nutrients intake from diet and dietary products and supplements. Both in cases and controls, the average intake of protein and fat, especially SFA, expressed in percentage of the total energy intake (TEI) was higher than NOSP, while the average consumption of carbohydrate and dietary fiber was lower than NOSP. The cholesterol intake was also lower than the NOSP. In particular, protein intake was higher than NOSP in 60.7% of the sample, and fat intake in 94.2%. Carbohydrate intake was lower than NOSP in 99.0%, and dietary fiber in 93.0%. There were no significant differences in any of these variables between cases and controls. Nevertheless, the percentage of cases whose consumption of SFA and cholesterol did not comply with NOSP was higher compared to controls (SFA: 85.7% vs. 67.5%, *p* < 0.001; cholesterol: 35.4% vs. 25.0%, *p* < 0.01). After Bonferroni adjustment for multiple testing (*p* = 0.05 ÷ 31), the percentage of cases whose consumption of SFA did not comply with NOSP was significantly higher than those of controls (*p* = 2.31 × 10^−9^).

The average intake of vitamin B_1_, B_6_/protein, cholesterol and caffeine was higher in cases than controls (*p* < 0.05), while vitamin B_2_ and the calcium:phosphorus ratio (Ca:P) were higher in controls than cases (*p* < 0.01). These differences between cases and controls did not withstand Bonferroni correction, even if they did meet nominal significance. The percentage of cases whose vitamin B_2_ intake did not comply with NOSP was higher than that of controls (7.1% vs. 2.9%, *p* < 0.05). Concerning the average consumption of caffeine, this was lower than the recommended limit in both cases and controls (400 mg/d).

On the other hand, the percentage of EAR (from diet and dietetic products and supplements) for calcium, iron and vitamin B_2_ was lower in cases than controls (*p* < 0.05), while the percentage of EAR for vitamin B_1_ and B_6_ was lower in controls than cases (*p* < 0.05) (Table 4). However, all these differences did not reach the Bonferroni corrected level of statistical significance. The nutrients with the lowest proportions of subjects with intakes below EAR were vitamin B_3_ (4.8%), iron (9.7%), vitamin B_12_ (21.4%) and phosphorus (23.7%), and there were no significant differences between cases and controls in these percentages. The percentage of the total sample (cases and controls) that exceeded the UL was 18.8% for magnesium (of which 1% used supplements and/or dietetic products), 37.4% for sodium and 47.7% for vitamin B_3_ (of which 1.6% used supplements and/or dietetic products), there were no significant differences between cases and controls. In the cases group, 0.9% exceeded the UL value for vitamin B_6_.

Finally, the average mineral score was 20.2 ± 3.4 with a range between 14 and 28, without significant differences between cases and controls. The distribution in tertiles of the mineral score showed a greater proportion of controls (76%) than cases (24%) in the second tertile (*p* < 0.001), and a greater proportion of cases than controls in the third tertile (59.6% vs. 40.0%, *p* < 0.001; respectively). These differences remained significant after Bonferroni adjustment for multiple testing (*p* = 0.05 ÷ 31); the first one was 4.98 × 10^−38^, and the second one 8.85 × 10^−7^.

## 4. Discussion

This study was successful in obtaining a sample of cases and controls from the population-based BCSP of the Basque Health Service (Osakidetza), all of whom agreed to participate in the full study protocol (self-reported data, clinical databases and genotyping). More men than women participated (1.96:1.0), and they were mostly elderly people (average age in cases = 61.5; and in controls = 61.1 years), which was consistent with previous literature on BCSP of the Osakidetza/Basque Health Service [74]. Although the average participation rate in this BCSP was higher in women than in men (70.9% vs. 65.6%), the proportion of CRC diagnosed was higher in men than in women (4.8% vs. 2.1%) [74].

With regard to the characteristics of the sample studied, the prevalence of overweight/obesity was higher in cases than controls. This result is in agreement with previous studies [75,76,77] that have confirmed that obesity is associated with an increased risk of CRC. Even though the biological mechanisms underlying the association between body-fat in excess and CRC remain unclear [78], evidence seems to support the important role of metabolic syndrome, insulin resistance [79], systemic inflammation and immunity [80], microbial dysbiosis [81], and certain genetic factors especially in early onset CRC [82,83]. Elucidating the mediating role of these factors in obesity-induced CRC should be very useful in the prevention and treatment of this type of cancer. In addition to the direct contribution of obesity to CRC risk, body-fat in excess, in turn, could be associated with other risk factors for CRC, such as unhealthy diet and sedentary lifestyle [84,85]. Notably, we also observed a slightly higher proportion of controls whose main daily activity was sedentary compared to cases, but this result could be influenced by a greater awareness of the associations between diseases and lifestyle factors among cases.

Regarding the diet, no significant differences were found for the food group studied, except for the frequency of use of salt added to cooking that was significantly higher in cases than controls. In other case-control studies, a positive association between sodium intake and CRC was also observed [86]. In any case, our average intake of sodium from SFFQ was similar in cases as controls, probably due to the difficulty to estimate this intake from self-reported data on salt added [87]. From the nutritional point of view, the diet of participants, both cases and controls, was characterized by high intakes of protein, fat and SFA, and low intakes of carbohydrates and dietary fiber. Thus, it was a western diet pattern. This dietary pattern has been associated before with an elevated CRC incidence [88,89].

Moreover, the percentage of cases whose consumption of SFA and cholesterol did not comply with NOSP was higher than controls. This result is in agreement with those reported by other authors that have observed a higher CRC risk among subjects with high intake of both SFA and cholesterol (highest vs. lowest) [90]. Arafa et al. [91] also reported a higher intake of saturated fats and cholesterol among CRC-diagnosed subjects as compared to controls. The mechanisms involved in the influence of fat on the colorectal carcinogenesis are complex and appear to be related to its effect on the insulin-signal pathway and the c-Hun N-terminal kinase pathway that promote the colonic cell proliferation [92].

On the other hand, in the present study, we have not found a higher intake in controls than in cases of protective factors associated with a decrease in CRC risk according to the scientific literature, such as calcium, magnesium, fiber diet, vitamin D, B_6_ or regular use of certain drugs [9]. However, the average intake of vitamin B_2_ and the Ca:P was higher in controls than cases, and neither reached the Bonferroni-corrected level of statistical significance. Some studies have indicated before that vitamin B_2_ intake is inversely associated with CRC risk [93]. Although this vitamin has received less attention than other ones as a protective factor of epithelial cancers (including CRC), the interest in vitamin B_2_ is increasing due to the role of flavins in folate metabolism and the possible synergistic protective effect between these two vitamins for cancer [94]. With respect to Ca:P, Boutron et al. [95] reported a case–control study in which they analyzed the possible association between this ratio and colorectal carcinogenesis and found positive associations, but they did not observe any modulation by P intake of the association between dietary Ca intake and CRC.

On the contrary, the hypothesis that higher intakes of minerals with colon anti-carcinogenic effects combined with lower intakes of those minerals with pro-carcinogenic effects may be associated with lower CRC risk was not supported. However, after Bonferroni correction for multiple comparisons, a greater proportion of controls than cases positioned in the second tertile of the minerals score, in contrast to what has been observed in the third tertile.

The main strength of this study is the fact that information is provided based on a standardised protocol including not only dietary and genetic factors but also other possible determinants of CRC such as health determinants and weight status among others. Another strength of this study compared to others [23,24,25] is that colonoscopy was used as a diagnosis criterion to identify the cases in order to avoid false positives and negatives. However, there are some limitations that should be mentioned. First, recall bias inherent in a case–control study design cannot be ruled out. Second, self-reported data could be subject to measurement errors and the problem of food omissions due to memory failure and under-reporting of unhealthy habits among disease subjects. However, previous validation studies indicate that the self-reported dietary information is reported with sufficient accuracy for use in epidemiology analysis [96]. Even though data on lifestyles (including diet) were recorded retrospectively—that is, the questions referred to behaviors before participating in the BCSP—it should be also noted that dietary changes are usually modest after participating in the BCSP due to a lack of information and personalized advice [97,98]. Finally, to avoid selection bias of controls, we obtained controls from the same BCSP and in the same period as cases; thus, it was confirmed that they did not suffer from CRC by colonoscopy.

## 5. Conclusions

This study provides valuable data for analyzing the complexity of gene–diet interaction in relation to CRC in a sample from a screening program. These data include not only lifestyle and genetic determinants of CRC risk but also demographic, socio-economic data, weight status, perceived quality of life and stress, use of drugs related to decreasing CRC risk and lifestyle changes after participating in the BCSP. Thus, this research provides valuable data for analyzing the determinants of this pathology and for designing prevention strategies. The authors hope that this report could help other researchers replicate this survey in other populations in order to easily and accurately compare their results. However, it is worth noting that some questionnaires include culturally sensitive topics such as dietary habits and should be adapted to and validated in the population of interest. In addition, the results presented in this manuscript allow us to conclude that cases were more likely than controls to have overweight/obesity, a higher frequency of consumption of salt added for cooking, a lower intake of vitamin B_2_ and Ca:P, and not to have adequate intakes of SFA and cholesterol. Thus, some environmental factors, such as weight status and dietary components, could influence the etiology of CRC in this population.

## Figures and Tables

**Table 1:** Overview of the measurements and variables collected in this survey.

Method	Measure of Interest	Variables ^a,b^
Self-reported data	Weight status	**Self-Reported Weight (kg) and Height (cm).** **BMI**
	Demographic data	**Age, sex.**
	Socio-economic data	Educational attainment, economic activity, last employment, total number of co-residents per household and total number of rooms, excluding the kitchen and bathrooms. HCI.
	Lifestyle	**PA** (main daily activity), *PE* (at least 20 minutes per session), smoking habit (yes or no, age at start, number of cigarettes per day, years without smoking), and alcohol consumption (frequency and type of alcohol, these items were included in the SFFQ). **Dietary habits: SFFQ, dietetic products and nutrient supplements consumption.** **Energy intake, macro/micronutrients** (based on food composition tables), **adequacy of energy and nutrients intake** (percentage of the EER, NOSP, AI and UL), **mineral score** and diet quality index (HEISD and MDS). Adherence to guidelines for CRC prevention (PA/PE, dietary fiber, red meat and processed meat, alcoholic beverages and BMI) (subsample randomly selected).
	Quality of life and stress	**Perceived quality of life and stress.**
	Drugs	**Antiplatelet, anticoagulants and HRT (females).**
Clinical databases	Date of participation in the *BCSP*	Date of the iFOBT Time spent between iFOBT and participation in this survey.
	Diagnosis and treatment (cases)	Pathological staging, location of the cancer, differentiation, tumor grade and treatments (type and data of surgery, radiation therapy and/or chemotherapy). Time spent between treatments and participation in this survey.
	Socio-economic level	DI
	Health status (specifically health resource consumption)	PRM
Phone interviews	Lifestyle changes after participating in the BCSP (subsample)	PA/PE, food consumption (vegetables, fruits, whole cereals, red meat, processed meat, alcoholic drinks) and smoking habits.
Genotyping	48 SNPs of susceptibility 82 SNPs that could be associated with food groups or dietary factors and gene–diet interactions	

AI: adequate intakes; BCSP: bowel cancer screening program; BMI: body mass index; DI: deprivation index; EER: estimated energy requirements; HCI: household crowding index; HEISD: Healthy Eating Index for Spanish Diet; HRT: hormone replacement therapy; iFOBT: immunochemical fecal occult blood test; MDS, MedDietScore; NOSP: nutritional objectives for the Spanish population; PA: physical activity; PE: physical exercise; PRM: predictive risk modeling; SFFQ: short food frequency questionnaire; SNP: single nucleotide polymorphism; UL: daily tolerable upper limits. ^a^ Text in italics corresponds to data derived from direct measurements. ^b^ Results presented in this manuscript are highlighted in bold.

**Table 2:** Demographic data, weight status, lifestyle, drugs use, quality of life and stress level in cases and controls studied.

Variables	Cases (*n* = 308)	Controls (*n* = 308)	*p* ^a^
Sex, % of men	66.2	66.2	
Age, y, mean ± SD	61.5 ± 5.2	61.1 ± 5.5	0.093
BMI, kg/m^2^, mean ± SD	27.5 ± 4.4	26.8 ± 4.4	**0.049**
Overweight/obesity, %	67.5	58.1	**0.015**
Main daily activity, %			
Sitting	31.8	40.1	
Standing	29.8	29.9	
Walking	34.1	28.6	
Activities that demanded great physical effort	4.3	1.3	0.079
Alcohol consumption, SDU, mean ± SD	0.9 ± 1.0	0.8 ± 0.9	0.682
Non-compliance with recommendations, %	13	12	0.715
Dietetic products and supplement use ^b^, %	9.7	13	0.306
Among those who consumed dietetic products and/or supplements			
Dietetic products use, %	26.1	21.7	0.249
Milk/dairy ^d^	28.6	16.7	
Vegetable drinks ^e^	71.4	66.6	
Iodized salt	-	16.7	1
Nutrient supplements, %	73.9	13	0.306
Vitamins	19.4	15.9	
Minerals	29	27.3	
Complex vitamin-mineral products	12.9	13.6	
n-3 PUFA	16.1	22.7	
n-6 PUFA	6.5	-	
Fiber	16.1	20.5	0.701
Drugs, %			
Antiplatelet	18.5	13.7	0.038
Anticoagulants	1.3	1.3	0.359
HRT (females) ^f^	16	16.7	0.585
QoL ^g^, mean ± SD	71.5 ± 14.0	69.8 ± 15.2	0.141
Stress level ^g^, mean ± SD	40.4 ± 26.4	41.1 ± 24.8	0.846

BMI: body mass index; HRT: hormone replacement therapy; PUFA: polyunsaturated fatty acids; QoL: quality of life; SD: standard deviation; SDU: standard drink unit. ^a^ Differences between cases and controls. Significant results are highlighted in bold. ^b^ Percentage calculated on the number of cases and controls. ^c^ This question presented multiple answers. Percentage based on the number of answers obtained in each item. ^d^ Enriched with calcium, omega-3, lactose-free milk. ^e^ Rice, oat or soy drinks. ^f^Percentage of females. ^g^ To assess the QoL and perceived stress an analog linear scale with a range from 0 to 100 was used.

**Table 3:** Daily energy and nutrients intake (from diet and dietetic products and supplements) in cases and control studied.

Daily Intake from Diet and Dietetic Products and Supplements	Cases (*n* = 308)	Control (*n* = 308)	*p* ^a^
	Mean ± SD	Mean ± SD	
Energy, kcal/d	1774.3 ± 388.0	1743.1 ± 390.9	0.205
Macronutrients			
Protein, % TEI	15.7 ^b^ ± 2.3	16.1 ± 7.1	0.681
NOSP, 10–15% TEI			
Carbohydrates,% TEI	36.2 ± 4.9	36.7 ± 5.6	0.277
NOSP, 50–60% TEI			
Fat, %TEI	42.5 ± 4.5	42.1 ± 5.1	0.256
NOSP, <30–35%			
SFA, % TEI	12.7 ± 2.5	12.4 ± 2.8	0.179
NOSP, <7–8% TEI			
MUFA, % TEI	19.6 ± 2.7	19.7 ± 2.8	0.631
NOSP, 20% TEI			
PUFA, % TEI	6.6 ± 2.0	6.3 ± 1.7	0.183
NOSP, 5% TEI			
Minerals and electrolytes			
Calcium, mg	759.4 ± 238.0	780.6 ± 227.0	0.225
NOSP, 800–1000 mg			
Phosphorus, mg	1220.2 ± 314.5	1207.9 ± 297.6	0.648
Ca:P ^c^	0.6 ± 0.1 ^b^	0.6 ± 0.1 ^b^	0.009
NOSP, 1,3:1			
Iron, mg	14.1 ± 3.9	14.4 ± 4.2	0.765
Magneisum, mg	263.1 ^b^ ± 73.7	263.8 ± 63.1 ^b^	0.760
Potassium, mg	2616.0 ± 615.3	2627.7 ± 610.2	0.954
Iodine, µg	88.8 ± 39.9	87.3 ± 24.7	0.961
NOSP, 150 µg			
Sodium ^d^, mg	1950.8 ± 1041.4	1820.1 ± 1004.3	0.081
NOSP, <2000 mg/d			
Selenium, µg	88.3 ± 24.3	87.3 ± 24.4	0.682
Copper, mg	1.0 ± 0.3	1.0 ± 0.3	0.568
Zinc, mg	9.4 ± 2.8	9.3 ± 2.9	0.375
Vitamins			
Vitamin B_1_, mg/1000 kcal	1.0 ± 7.6 ^e^	0.6 ± 0.2	0.003
NOSP, 0.4 mg/1000 kcal			
Vitamin B_2_^f^, mg/1000 kcal	0.9 ± 0.2	0.9 ± 0.2	0.002
NOSP, 0.6 mg/1000 kcal			
Vitamin B_3_, mg/1000 kcal	17.1 ± 3.3	17.2 ± 3.3	0.117
NOSP, 6.6 mg/1000 kcal			
Vitamin B_6_ (mg)/protein (g)	0.04 ± 0.2	0.03 ± 0.01	0.020
NOSP, >0.02 vitamin B_6_ (mg)/protein (g)			
Folate, µg	267.2 ± 80.1	273.3 ± 76.5	0.406
NOSP, >300–400 µg			
Vitamin B_12_, µg	6.7 ± 28.5 ^e^	4.9 ± 1.7	0.094
Vitamin C, mg	149.3 ± 66.0	147.8 ± 59.9	0.611
Vitamin A, µg	532.4 ± 206.9	522.0 ± 181.4	0.420
Vitamin D, µg	2.1 ± 1.0	2.3 ± 1.9	0.799
NOSP (>50 y old), 10 µg			
Vitamin E, mg	0.6 ± 0.1	0.6 ± 0.1	0.139
NOSP, >0.4 vitamin E (mg)/PUFA (g)			
Others			
Cholesterol, mg	274.7 ^b^ ± 96.7	256.2 ± 95.3	0.019
Fiber, g	19.9 ± 6.5	20.1 ± 6.0	0.459
Caffeine, mg	15.1 ± 9.7	13.8 ± 11.7	0.025

Ca:P: calcium:phosphorus ratio; MUFA: monounsaturated fatty acids; NOSP, Nutritional objectives for the Spanish population; PUFA: polyunsaturated fatty acids; SD, standard deviation; SFA: saturated fatty acids; TEI: total energy intake. ^a^ Differences between cases and controls. ^b^ This variable followed a normal distribution. ^c^ Ca:P was 0.62 ± 0.12 in cases and 0.65 ± 0.13 in controls. ^d^ Sodium from foods; does not include the amount of salt added. ^e^ This high SD is due to the use of nutritional supplements by one of the cases. ^f^ Vitamin B_2_ intake per 1000 kcal was 0.86 ± 0.23 in cases and 0.92 ± 0.23 in controls.

**Table 4:** Energy and nutrients intake (from diet and dietetic products and supplements) expressed as a percentage of the estimated energy requirement (EER), the estimated average requirements (EAR) or adequate intakes (AI) in cases and controls studied.

% of Energy Requirement Estimated, EARs or AI	Cases (*n* = 308)	Control (*n* = 308)	*p* ^a^
	Mean ± SD	Mean ± SD	
Energy	114.4 ± 32.7	107.0 ± 31.3	0.056
Minerals and electrolytes			
Calcium	49.7 ± 11.1 ^b^	52.7 ± 13.4 ^b^	0.025
Phosphorus	118.9 ± 18.1	120.1 ± 18.5	0.469
Iron	142.5 ± 35.7	147.5 ± 36.4	0.044
Magnesium	47.5 ± 13.2	48.1 ± 12.0	0.094
Potassium	55.7 ± 13.1	55.9 ± 13.0	0.081
Iodine	53.2 ± 22.9	53.0 ± 10.5	0.236
Sodium	150.1 ± 80.1	140.0 ± 77.3	0.954
Selenium	111.9 ± 24.6	112.3 ± 24.6	0.598
Copper	80.6 ± 13.4	81.1 ± 13.1	0.360
Zinc	64.0 ± 19.5	64.1 ± 18.7	0.938
Vitamins			
Vitamin B_1_^d^	110.7 ± 847.4	66.5 ± 19.4	0.003
Vitamin B_2_	84.6 ± 25.5	89.9 ± 24.8	0.002
Vitamin B_3_	147.4 ± 30.5	150.5 ± 29.0	0.121
Vitamin B_6_	110.8 ± 73.6	81.2 ± 19.6	0.010
Folate	47.8 ± 13.7	49.8 ± 13.1	0.060
Vitamin B_12_	185.2 ± 763.1 ^d^	139.2 ± 36.5	0.350
Vitamin C	124.0 ± 55.5	124.5 ± 51.7	0.865
Vitamin A	52.5 ± 19.9	52.4 ± 17.5	0.943
Vitamin D	11.9 ± 5.7	13.1 ± 11.9	0.956
Vitamin E	38.3 ± 16.3	35.7 ± 12.2	0.053

AI: adequate intakes; EAR: estimated average requirements; SD: standard deviation. ^a^ Differences between cases and controls. ^b^ This variable followed a normal distribution. ^c^ Nutrient intake expressed as a percentage of the AI (the rest of the nutrients were expressed as a percentage of the EAR). ^d^ This high SD is due to the use of nutritional supplements by one of the cases.

## Abbreviations

AI, adequate intake; ASRi, age-standardized incidence rate; BCSP, bowel cancer screening program; BMI, body mass index; Ca:P, calcium:phosphorus ratio; CRC, colorectal cancer; DI, deprivation index; EAR, estimated average requirements; EER, estimated energy requirement; GQ, general questionnaire; GRS, genetic risk score; HEISD, Healthy Eating Index for Spanish Diet; iFOBT, immunochemical quantitative test; L, level; MDS, MedDietScore; MUFA, monounsaturated fatty acids; NOSP, nutritional objectives for the Spanish population; PA, physical activity; PE, physical exercise; PRM, predictive risk modeling; PUFA, polyunsaturated fatty acids; SFFQ, short frequency questionnaire; SDU, standard drink unit; SFA, saturated fatty acids; SNP, single nucleotide polymorphism; Q, quintile; QoL, quality of life; TEI, total energy intake; WCRF/AICR, World Cancer Research Fund/American Institute for Cancer Research.
